# Small-angle neutron scattering study of mesoscale magnetic disordering and skyrmion phase suppression in the frustrated chiral magnet Co_6.75_Zn_6.75_Mn_6.5_ 1

**DOI:** 10.1107/S1600576722007403

**Published:** 2022-09-14

**Authors:** Jonathan S. White, Kosuke Karube, Victor Ukleev, P. M. Derlet, R. Cubitt, C. D. Dewhurst, A. R. Wildes, X. Z. Yu, H. M. Rønnow, Yoshinori Tokura, Yasujiro Taguchi

**Affiliations:** a Laboratory for Neutron Scattering and Imaging, Paul Scherrer Institute, Villigen, CH-5232, Switzerland; b Center for Emergent Matter Science (CEMS), RIKEN, Wako, 351-0198, Japan; c Condensed Matter Theory Group, Paul Scherrer Institute, Villigen, CH-5232, Switzerland; d Institut Laue–Langevin, 71 avenue des Martyrs, CS 20156, Grenoble, 38042 Cedex 9, France; e Laboratory for Quantum Magnetism (LQM), Institute of Physics, École Polytechnique Fédérale de Lausanne (EPFL), Lausanne, CH-1015, Switzerland; f Department of Applied Physics, University of Tokyo, Bunkyo-ku, 113-8656, Japan; g Tokyo College, University of Tokyo, Bunkyo-ku, 113-8656, Japan; University of Luxembourg

**Keywords:** small-angle neutron scattering, skyrmions, chiral magnets, frustration, magnetic disorder, diffuse scattering

## Abstract

Co–Zn–Mn chiral cubic magnets display versatile magnetic skyrmion phases, including equilibrium phases stable far above and far below room temperature, and the facile creation of robust far-from-equilibrium skyrmion states. In this system, compositional disorder and magnetic frustration are key ingredients that have profound effects on the chiral magnetism. Reported here are studies of the magnetism in Co_6.75_Zn_6.75_Mn_6.5_ by magnetometry, small-angle neutron scattering (SANS), magnetic diffuse neutron scattering and Lorentz transmission electron microscopy (LTEM). While features in magnetometry and LTEM often give standard indications for skyrmion formation, they are not readily observed from the measurements on this system. Instead, skyrmion lattice correlations are only revealed by SANS, and they are found to form an orientationally disordered structure in a minority fraction of the sample. The majority fraction of the sample always displays orientationally disordered helical spin correlations, which undergo further disordering along the radial direction on cooling below the critical temperature (*T*_c_ ≃ 102 K). The near-complete suppression of the skyrmion phase, and the process of disordering on cooling, are attributed to competing magnetic interactions that dominate over the ferromagnetic interaction expected to favour chiral magnetism in this system. These competing interactions start to develop above *T*_c_ and become further enhanced towards low temperatures. The present observations of co-existing and disordered magnetic correlations over multiple length scales are not unique to Co_6.75_Zn_6.75_Mn_6.5_ but are seemingly common to the family of Co–Zn–Mn compounds with finite Mn, and their accurate description presents a challenge for theoretical modelling. In addition, this study highlights a need for neutron instrumentation capable of the comprehensive measurement of magnetic correlations over expanded ranges of momentum transfer in such multiple-length-scale magnets.

## Abstract 2

In the frustrated chiral magnet Co_6.75_Zn_6.75_Mn_6.5_, small-angle neutron scattering reveals that the mesoscale chiral magnetism displays strong disorder and the skyrmion phase is nearly entirely suppressed.

## 1. Introduction

Small-angle neutron scattering (SANS) is an established technique for scrutinizing mesoscale incommensurately modulated magnetic structures in solids (Mühlbauer *et al.*, 2019 ▸). Nowhere is this clearer than in the study of spiral, vortex, meron and skyrmion phases in various non-centrosymmetric magnets, ranging from the itinerant *B*20s MnSi (Mühlbauer *et al.*, 2009 ▸) and FeGe (Moskvin *et al.*, 2013 ▸), to the intermetallics Co–Zn–Mn (Tokunaga *et al.*, 2015 ▸), CeAlGe (Puphal *et al.*, 2020 ▸) and Y_3_Co_8_Sn_4_ (Takagi *et al.*, 2018 ▸), to the insulators Cu_2_OSeO_3_ (Seki *et al.*, 2012 ▸), GaV_4_Se_8_ (Bordács *et al.*, 2017 ▸) and VOSe_2_O_5_ (Kurumaji *et al.*, 2017 ▸), to name a few. In itinerant chiral cubic magnets, the antisymmetric Dzyaloshinskii–Moriya interaction (DMI) *D* arises from the spin–orbit interaction in the presence of broken space inversion, and competes microscopically with a dominant ferromagnetic exchange *J*. This competition leads to the formation of a long-period helical spiral ground state that modulates with a real-space period λ ∝ *J*/*D* along directions determined by magnetic anisotropy. Typical length scales for the helical modulation range from a few to a few hundred nanometres (Kanazawa *et al.*, 2017 ▸; Tokura & Kanazawa, 2021 ▸), lying within the range accessible by SANS. Such a length scale is also inherited by topological skyrmions that form out of helical order under a small magnetic field *H* and near the critical temperature *T*_c_. Since fluctuations are crucial for skyrmion phase stability (Mühlbauer *et al.*, 2009 ▸; Kruchkov *et al.*, 2018 ▸), the equilibrium skyrmion phase is correspondingly narrow in temperature span, being just a few percent of *T*_c_ wide directly below *T*_c_. Otherwise, the non-topological helical or field-induced helical cone (‘conical’) phases dominate the rest of the phase diagram (Kanazawa *et al.*, 2017 ▸; Tokura & Kanazawa, 2021 ▸).

**Figure 1:**
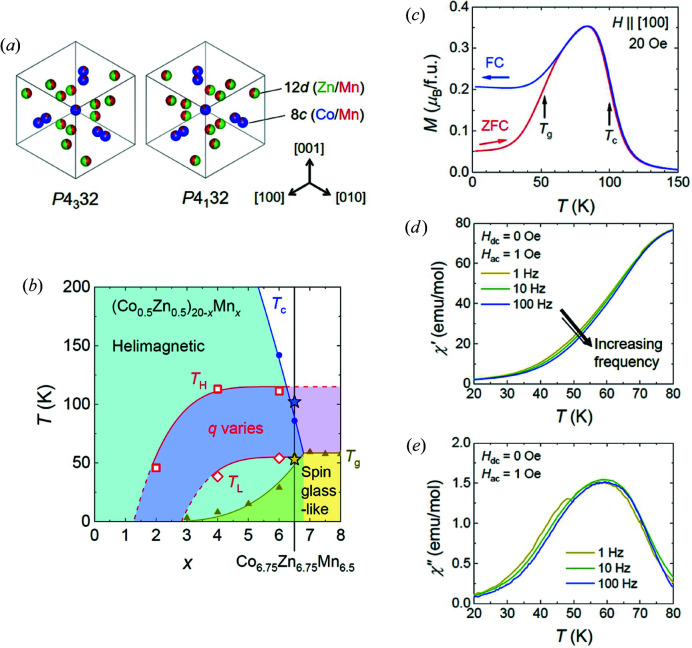
(*a*) The two β-Mn-type crystal enantiomers of Co_6.75_Zn_6.75_Mn_6.5_ viewed along the [111] direction. The 8*c* and 12*d* Wyckoff sites are coloured according to their average occupation of Co (blue), Zn (green) and Mn (red). (*b*) The low-temperature (*T*) versus Mn concentration *x* in (Co_0.5_Zn_0.5_)_20−*x*_Mn_*x*_, for 0 ≤ *x* ≤ 8, determined from magnetization measurements. Circle and triangle symbols denote magnetic transitions identified previously from measurements on polycrystalline samples (Karube *et al.*, 2018 ▸). Star symbols correspond to new data obtained from the Co_6.75_Zn_6.75_Mn_6.5_ crystals studied here. The shaded purple region denotes a *T* range for which the magnetization at *H* = 20 Oe decreases, and concomitantly the helical wavevector *q* observed by SANS increases, for decreasing *T*. The square (diamond) symbols indicate temperatures where *q* starts increasing (stops increasing) on cooling, as denoted by *T*_H_ (*T*_L_). (*c*) The temperature dependence of the field-cooled (FC) and zero-field-cooled (ZFC) d.c. magnetization in single-crystal Co_6.75_Zn_6.75_Mn_6.5_. (*d*), (*e*) The temperature and frequency dependence of, respectively, the real (χ′) and imaginary (χ′′) parts of the a.c. susceptibility of Co_6.75_Zn_6.75_Mn_6.5_ in the low-*T* range.

Recently, Co–Zn–Mn compounds have emerged as a novel class of skyrmion-hosting chiral magnets (Tokunaga *et al.*, 2015 ▸; Karube *et al.*, 2016 ▸, 2017 ▸, 2018 ▸, 2020 ▸; Henderson *et al.*, 2021 ▸). This system crystallizes in the β-Mn chiral cubic structure with either a *P*4_1_32 or *P*4_3_32 space group, and with 20 atoms distributed across the 8*c* and 12*d* Wyckoff sites [Fig. 1 ▸(*a*)] (Hori *et al.*, 2007 ▸; Xie *et al.*, 2013 ▸; Bocarsly *et al.*, 2019 ▸; Nakajima *et al.*, 2019 ▸). For the composition line (Co_0.5_Zn_0.5_)_20−*x*_Mn_*x*_ (0 ≤ *x* ≤ 20), both density functional theory calculations (Bocarsly *et al.*, 2019 ▸) and neutron diffraction show that magnetic Co preferentially occupies the 8*c* site, while magnetic Mn and non-magnetic Zn preferentially fill in on the 12*d* site (Bocarsly *et al.*, 2019 ▸; Nakajima *et al.*, 2019 ▸). For mixed composition samples, the simultaneous occupancy of both sites by just a single atomic species is never perfect, leading to inevitable compositional disorder. Nonetheless, the average β-Mn structure is maintained for all *x*, thus describing compositions ranging from Co_10_Zn_10_ to Mn_20_ or β-Mn itself (Karube *et al.*, 2018 ▸; Nakajima *et al.*, 2019 ▸).

Fig. 1 ▸(*b*) shows the low-temperature portion of a phase diagram that reports characteristic temperatures observed in magnetometry measurements versus Mn content *x*. The end member Co_10_Zn_10_ (*x* = 0) is a high-temperature chiral magnet, where Co spins on the 8*c* site order helically with a period λ ≃ 150 nm below the transition temperature *T*_c_ ≃ 420 K [beyond the upper temperature limit of Fig. 1 ▸(*b*)] and form an equilibrium skyrmion phase that is stable far beyond room temperature (Karube *et al.*, 2020 ▸). In this low-*x* limit, the general paradigm of DMI chiral magnetism observed in structurally simpler chiral cubic magnets like the *B*20s broadly applies (Kanazawa *et al.*, 2017 ▸).

Introduction of Mn leads to a rapid suppression of *T*_c_ to far below room temperature [Fig. 1 ▸(*b*)] and a decrease in the helical periodicity near *T*_c_ – and hence in skyrmion size – from λ ≃ 130 nm in Co_9_Zn_9_Mn_2_ to λ ≃ 110 nm in both Co_8_Zn_8_Mn_4_ and Co_7_Zn_7_Mn_6_ (Karube *et al.*, 2020 ▸). As indicated by the shaded purple region in Fig. 1 ▸(*b*), for fixed compositions 2 < *x* < 6, below *T*_c_, λ decreases by ∼40–50% upon cooling between two temperatures *T*_H_ < *T* < *T*_L_. This manifests as an increase in the magnitude of the helical propagation vector *q* (= 2π/λ) measured by SANS, and indicates a reduction in the ratio of *J*/*D*. This behaviour is interpreted as arising due to an onset of antiferromagnetic-like correlations between Mn moments on cooling that affect the ferromagnetic interactions and DMI between Co (Karube *et al.*, 2018 ▸, 2020 ▸). At lower temperatures below *T*_L_, a reentrant spin glass transition *T*_g_ is observed for 3 < *x* < 6, which is known to involve the 8*c* helical spin correlations (Karube *et al.*, 2020 ▸). The origin of the spin-glass-like properties can be traced from the physics of the other end member, β-Mn (*x* = 20), a well known elemental spin liquid (Nakamura *et al.*, 1997 ▸). In its pure form, β-Mn displays no spin glass transition, and the spin liquid property is due to the strong geometric frustration of antiferromagnetically coupled local Mn moments residing on the hyper-kagomé-coordinated 12*d* site. Spin-glass-like transitions emerge in lightly doped β-Mn alloys, due to combined geometric frustration and compositional disorder (Nakamura *et al.*, 1997 ▸; Stewart *et al.*, 2002 ▸, 2008 ▸, 2010 ▸; Stewart & Cywinski, 2009 ▸). In the presently studied composition line, no helical transition is seen for 6.5 < *x* < 19, and instead only a spin-glass-like transition *T*_g_ is observed (Karube *et al.*, 2018 ▸).

Overall, the phase diagram of the present class of Co–Zn–Mn chiral magnets combines various key ingredients in a single system, namely mainly Co DMI chiral magnetism on the 8*c* site, magnetic frustration mainly due to antiferromagnetic correlations between Mn spins on the 12*d* site, magnetic anisotropy that varies with *T* and *x* (Preißinger *et al.*, 2021 ▸), and compositional disorder. Tuning the composition controls the relative influence of each aspect and has thus far led to a series of novel findings. These include the observation of equilibrium skyrmion phases near *T*_c_, both far above and far below room temperature (Tokunaga *et al.*, 2015 ▸), and the facile disorder-assisted creation of supercooled metastable skyrmion states (Karube *et al.*, 2016 ▸, 2017 ▸, 2018 ▸, 2020 ▸; Morikawa *et al.*, 2017 ▸). These metastable skyrmion states are practically infinitely long lived, survive over the majority of the phase diagram, and display novel and reversible coordination transformations that preserve the topological charge carried by the skyrmions. Finally, in Co_7_Zn_7_Mn_6_ the chiral magnetism and magnetic-frustration-induced fluctuations conspire to stabilize a second field-induced equilibrium skyrmion phase for temperatures just above *T*_g_ (Karube *et al.*, 2018 ▸; Ukleev *et al.*, 2021 ▸).

In this study we explore the helical, skyrmionic and short-range magnetic correlations in a new composition Co_6.75_Zn_6.75_Mn_6.5_ with *x* = 6.5 by SANS, magnetometry, magnetic diffuse neutron scattering and Lorentz transmission electron microscopy (LTEM) measurements. As seen in Fig. 1 ▸(*b*), the transitions at *T*_c_ and *T*_g_ are closer together for this composition compared with 3 < *x* < 6, indicating that magnetic frustration and disorder compete more strongly with DMI chiral magnetism in this system. At the same time, in terms of a reduced temperature Δ*T*/*T*_c_, the thermal extent of the equilibrium skyrmion phase has been found to grow progressively with *x* from 0.016 to 0.031 to 0.050 to 0.147 for *x* = 0, 2, 4 and 6, respectively (Karube *et al.*, 2020 ▸), indicating an enhanced stability. Moreover, for *x* = 6, a second equilibrium skyrmion phase is observed far below *T*_c_ (Karube *et al.*, 2018 ▸; Ukleev *et al.*, 2021 ▸), in accord with an enhanced stability of skyrmions. Therefore, it is important to understand how the helical magnetism and skyrmion phase stability evolve with *x* in the presence of competing interactions.

Here, we show experimentally that, for *x* = 6.5, the effects of magnetic frustration and disorder are so strong that they almost completely suppress the formation of the skyrmion phase near *T*_c_. Instead, SANS shows that the mesoscale spin correlations in the majority of the sample are dominated by strong magnetic disorder across the phase diagram. SANS nonetheless reveals that a ∼10% minority fraction of the sample does undergo a standard field-induced transition to a regime of skyrmion correlations near to *T*_c_ with the thermal window of stability *ΔT*/*T*_c_ ≃ 0.16. However, due to the generally strong magnetic disorder in the sample, the skyrmion correlations revealed by SANS are not clearly detectable by either magnetometry or LTEM. We discuss the results in the context of magnetic disorder induced on the 8*c* site through coupling with geometrically frustrated inter­actions on the 12*d* site. The important role of magnetic frustration is shown through direct measurement of short-range magnetic correlations in Co_6.75_Zn_6.75_Mn_6.5_ by diffuse neutron scattering, from which characteristic couplings between Mn moments on 12*d* sites are estimated.

## 2. Experimental

### 2.1. Sample preparation

**Figure 2:**
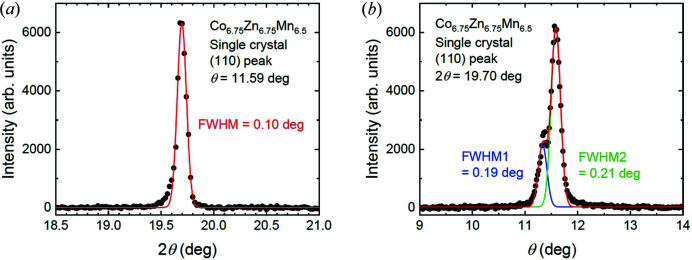
X-ray diffraction measurements of the (110) structural peak in a Co_6.75_Zn_6.75_Mn_6.5_ single crystal. (*a*) A 2θ scan of the (110) peak fitted by a single Gaussian line shape. (*b*) A rocking curve (*i.e.* θ scan) at fixed 2θ. The intensity distribution is fitted by two Gaussian line shapes.

**Figure 3:**
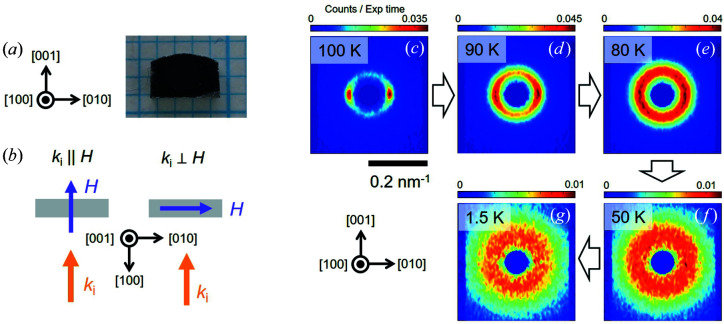
(*a*) A photograph and the crystal orientation of the Co_6.75_Zn_6.75_Mn_6.5_ single crystal studied by SANS. (*b*) Schematic diagrams of the two experimental geometries studied by SANS. In each geometry the neutron beam direction (**k**_i_) is normal to the largest face of the plate-like sample. The geometries differ according to the direction of the magnetic field **H**, with **H** ∥ **k**_i_ on the left and **H** ⊥ **k**_i_ on the right. (*c*)–(*g*) SANS patterns measured during ZFC at 100, 90, 80, 50 and 1.5 K, respectively. The intensity scale of the colour plot varies in each panel. The crystal orientation indicated for the SANS patterns in panels (*c*)–(*g*) is the same as for panel (*a*).

Bulk single crystals of Co_6.75_Zn_6.75_Mn_6.5_ were prepared by the Bridgman method. Single crystals were identified using X-ray Laue diffraction and their structural quality characterized at room temperature using Cu *K*α radiation from a laboratory X-ray diffractometer (Rigaku, RINT-TTR III). Fig. 2 ▸ shows typical diffraction data obtained from the (110) structural peak of one crystal extracted from the growth. According to the 2θ scan shown in Fig. 2 ▸(*a*) and the rocking scan shown in Fig. 2 ▸(*b*), the crystals display good structural quality with a finite mosaicity of less than 1°. Thus in the magnetic neutron scattering measurements that follow, the mosaic spread of the crystal can be considered unimportant with respect to the broad and diffuse magnetic scattering features observed. Further crystals were identified and cut from the same growth into regular-shaped platelets for both bulk magnetic and SANS measurements [Fig. 3 ▸(*a*)]. The chemical composition was examined by an energy-dispersive X-ray (EDX) spectrometer (Bruker XFlash6) equipped with a scanning electron microscope (JEOL JSM-6701F). The composition determined by EDX is Co_6.73 (1)_Zn_6.67 (2)_Mn_6.60 (2)_, close to the nominal composition.

Polycrystalline Co_6.75_Zn_6.75_Mn_6.5_ for neutron diffuse scattering measurements was synthesized from pure Co, Zn and Mn metals with nominal concentrations. The metals were sealed in evacuated quartz tubes, heated at 1473 K for 12 h, cooled to 1173 K at 1 K min^−1^, annealed for 24 h, and cooled down to room temperature (293 K) at 1 K min^−1^. Phase purity with the β-Mn-type structure was confirmed using X-ray diffraction.

For the LTEM measurement, a thin plate sample with a (001) surface and thickness ∼150 nm was prepared from a bulk single-crystal sample using a focused Ga ion beam.

### 2.2. Magnetometry measurements

Direct-current (d.c.) magnetization data for single-crystal Co_6.75_Zn_6.75_Mn_6.5_ were acquired using the vibrating-sample magnetometer mode of a superconducting quantum interference device magnetometer (Quantum Design MPMS3). Alternating-current (a.c.) susceptibility measurements were carried out using the a.c. measurement mode of the MPMS3. Both the static magnetic field and the a.c. excitation field (1 Oe) were applied along a cubic axis. The a.c. frequency was chosen to be 193 Hz except for frequency-dependent measurements.

### 2.3. Neutron scattering measurements

SANS measurements on a 57.2 mg single-crystalline sample of Co_6.75_Zn_6.75_Mn_6.5_ were performed on the D33 beamline at the Institut Laue–Langevin (ILL), Grenoble, France (White *et al.*, 2017 ▸, 2019 ▸). Neutrons with a wavelength of either 8 or 10 Å were collimated over a distance of 12.8 m before the sample. The scattered neutrons were detected by a two-dimensional position-sensitive multidetector positioned 12.8 m behind the sample. Data were collected over a range of momentum transfer 0.02 < *q* < 0.4 nm^−1^ [*q* = (4π/λ_n_)sinθ, where θ is half the scattering angle and λ_n_ is the wavelength of the incident neutron beam].

For SANS the single-crystal sample was installed in a horizontal-field cryomagnet at the sample position on the beamline. As shown in Fig. 3 ▸(*b*), two experimental geometries were studied, namely **H** ∥ **k**_i_ and **H** ⊥ **k**_i_, where **k**_i_ is the incoming neutron wavevector which was chosen to be parallel to [100] for both geometries. In either geometry, the SANS measurements were done by collecting data as the cryomagnet and sample were rotated together around the vertical axis over the neutron beam access range of ±10° provided by the cryomagnet windows. Detector measurements obtained at a range of rotation angles were summed together to produce a single image showing the distribution of magnetic scattering. Unless otherwise stated, SANS data were obtained at each temperature and magnetic field after an initial zero-field cooling through the critical temperature ∼102 K, and a subsequent *H* ramp once at the target temperature. Further data obtained either in the paramagnetic regime at 120 K or in a field-polarized regime were used for background subtraction of the low-*T*/low-*H* data to leave just the magnetic SANS signal of interest. SANS data reduction and analysis were performed using the *GRASP* software developed at the ILL (Dewhurst, 2003 ▸).

Magnetic diffuse neutron scattering (MDNS) experiments on a 17.9 g polycrystalline sample of Co_6.75_Zn_6.75_Mn_6.5_ were carried out on the D7 beamline at the ILL (White *et al.*, 2018 ▸). An *xyz* polarization analysis allowed pure isolation of the magnetic scattering cross section from the total scattering (Stewart *et al.*, 2009 ▸). The sample was loaded into a double-walled Al container and installed in a standard Orange He cryostat. For the experiment, an incoming neutron wavelength of 3.1 Å was used, and data were collected over a range of momentum transfer 0.6 < *q* < 3.8 Å^−1^ (or equivalently 6 < *q* < 38 nm^−1^).

### 2.4. LTEM measurements

LTEM measurements were performed with a transmission electron microscope (JEOL JEM-2800). A magnetic field was applied perpendicular to the plate, *i.e.* parallel to [001], and its magnitude was controlled by tuning the objective lens current of the microscope.

### 2.5. Demagnetization calibration

For single-crystal measurements, the relative demagnetization factors between measurement configurations with **H** parallel to the sample plate (d.c. and a.c. susceptibility, and SANS in the **H** ⊥ **k**_i_ geometry) and **H** perpendicular to the sample plate (SANS in the **H** ∥ **k**_i_ geometry) are different. To cater for the difference in demagnetization, data presented throughout this article with **H** parallel to the sample plate have had the field scale calibrated higher so that *H*′ = *H* × *N*, where *N* = 3.0.

## 3. Results and discussion

### 3.1. Magnetometry measurements

Fig. 1 ▸(*c*) shows the temperature (*T*)-dependent magnetization (*M*) in single-crystal Co_6.75_Zn_6.75_Mn_6.5_. The data are obtained in a small magnetic field of 20 Oe, either on field cooling (FC) or on field warming after an initial zero-field cooling (ZFC). In the high-*T* region for both curves, *M*(*T*) clearly increases (decreases) on the FC (ZFC) process. A transition temperature at *T*_c_ ≃ 102 K between helical and paramagnetic regimes is estimated from the inflection point of *M*_ZFC_(*T*), though it is noted that the transition is smeared over a broad *T* range compared with lower-*x* compounds (Karube *et al.*, 2020 ▸). Below 80 K, *M*_FC_(*T*) falls with decreasing *T*, and a clear separation between the *M*_FC_(*T*) and *M*_ZFC_(*T*) curves at ∼65 K can be interpreted as the onset of spin-glass-like behaviour.

To investigate the low-*T* behaviour further, Figs. 1 ▸(*d*) and 1 ▸(*e*) show the temperature and frequency dependence of, respectively, the real (χ′) and imaginary (χ′′) parts of the a.c. susceptibility. A frequency dependence of χ′ and χ′′ is a common spin glass characteristic, though in the present sample it is modest and exists over a broad thermal range. At the same time, no thermally sharp spin glass transition is seen. Therefore, while the material displays some tendency towards glassy behaviour at low temperature, further investigations are needed to determine if the low-temperature regime can be properly allocated as a spin glass. Here, for consistency with previous work on lower-*x* compounds (Karube *et al.*, 2020 ▸), in Figs. 1 ▸(*b*) and 1 ▸(*c*) we use the label *T*_g_ to denote the temperature where *M*_ZFC_(*T*) shows an inflection point, and thus the temperature where spin-glass-like behaviour is most clearly inferred in the data. For the present compound *T*_g_ ≃ 53 K can be compared with ∼65 K, which is where the magnetization hysteresis vanishes.

Overall, the bulk magnetic measurements are hallmarked by transition features – labelled *T*_c_ and *T*_g_ – that are thermally smeared. This contrasts strongly with sharper transitions observed at lower *x*, including the nearby composition Co_7_Zn_7_Mn_6_ (*x* = 6), indicating comparatively stronger disorder of the magnetically correlated phases in the present *x* = 6.5 composition which is likely to be manifested in the low-*T* magnetic spin configurations.

### 3.2. SANS measurements of helical and skyrmion correlations

#### 3.2.1. Helical disordering during zero-field cooling

To shed more light on the magnetometry data, we next turn to SANS investigations of the mesoscale magnetic spin correlations in a Co_6.75_Zn_6.75_Mn_6.5_ single crystal. Figs. 3 ▸(*c*)–3 ▸(*g*) show SANS patterns collected at selected temperatures during ZFC. Just below *T*_c_ at 100 K, the pattern shown in Fig. 3 ▸(*c*) is composed of two peaks with propagation vectors aligned with the [010] direction co-existing with an isotropic ring of scattering intensity. The intensity ring is indicative of incommensurate helical spin correlations with full orientational disorder, while the peaks are more typical for an incommensurate helical order with finite orientational order. The alignment of a helical propagation vector with the cubic axis is consistent with that observed in other Co–Zn–Mn compounds with finite Mn content (Karube *et al.*, 2016 ▸, 2017 ▸, 2018 ▸, 2020 ▸), perhaps indicating that the sign of magnetic anisotropy in *x* = 6.5 is the same as that observed for 2 ≤ *x* ≤ 6 (Karube *et al.*, 2020 ▸; Preißinger *et al.*, 2021 ▸). According to the cubic symmetry, additional peaks would be expected in this scattering plane due to a helical domain with propagation vectors aligned with the [001] direction. The observation of only two peaks indicates a preferential helical domain selection effect, which may be due to a residual sample strain.

On cooling to lower *T*, the two peaks seen at 100 K quickly broaden azimuthally, so that by 80 K the full decay of the orientational order of all helical spin correlations in the sample is complete and the entire SANS intensity appears as a ring around the origin of reciprocal space. On cooling further to the base temperature of 1.5 K, the intensity of the ring becomes weaker compared with higher *T* and is distributed over an extended range in |*q*|, thus reflecting a reduction in the radial correlation length. At all temperatures, the correlation length normal to the detector plane determined by sample rotation-angle-dependent (*i.e.* rocking curve) measurements remains nearly angle independent (data not shown). Overall, the ZFC data show that the sample always displays mainly three-dimensionally disordered helical spin correlations, with a clear reduction in the radial correlation length taking place on cooling.

**Figure 4:**
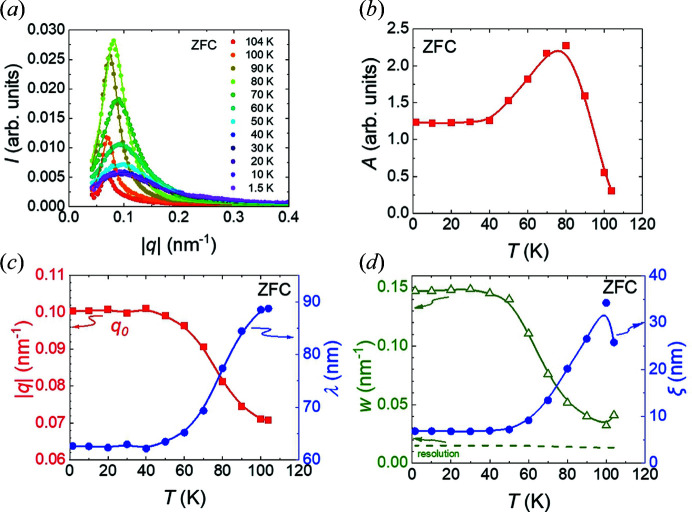
(*a*) The |*q*| dependence of the azimuthal-angle-averaged SANS intensity at several temperatures during ZFC. Data at each *T* are fitted with a Lorentzian function. (*b*) The ZFC temperature dependence of the integrated intensity *A* from the Lorentzian fits shown in panel (*a*). (*c*) The ZFC temperature dependence of the fitted peak position in |*q*|, namely *q*_0_ (square symbols), and the corresponding real-space periodicity λ = 2π/*q*_0_ (circle symbols). (*d*) The ZFC temperature dependence of the fitted Lorentzian peak FWHM *w* (triangle symbols) and the estimated radial correlation length ξ (circle symbols). Lines in panels (*b*), (*c*) and (*d*) are guides for the eye.

With Fig. 4 ▸, we analyse more quantitatively the magnetic SANS intensity observed during ZFC. Fig. 4 ▸(*a*) shows the radial |*q*| dependence of the SANS intensity integrated over all azimuthal angles. At all temperatures, the profiles are fitted well by a Lorentzian function, indicating the exponential decay of the radial correlation function with distance. The fitted function at each *T* is *I*(*q*) = *I*_0_ + (2*A*/π){*w*/[4(*q* − *q*_0_)^2^ + *w* ^2^]}, where *I*_0_ is a fitted constant background, *A* is the fitted integrated intensity of the peak, *w* is the fitted peak FWHM and *q*_0_ is the fitted peak centre. The temperature dependence of parameters *A*, *w* and *q*_0_ is shown in Figs. 4 ▸(*b*)–4 ▸(*d*).

From Fig. 4 ▸(*b*) we observe the integrated intensity *A* to rise initially on cooling below *T*_c_, before decreasing over the range from ∼80 to ∼40 K and then remaining approximately constant down to 1.5 K. The reduction in *A* over the intermediate temperature range largely coincides with significant enhancements of both *q*_0_ [Fig. 4 ▸(*c*)] and *w* [Fig. 4 ▸(*d*)]. Finally, all fitted quantities stop evolving with temperature after cooling below ∼40 K, consistent with being in a spin-glass-like regime.

The observed increase in *q*_0_ on cooling implies a reduction in the helical periodicity λ = 2π/*q*_0_, from 89 (1) nm near to *T*_c_ to 62 (1) nm by the base temperature. Qualitatively similar temperature dependences in *A* and *q*_0_, as observed in Fig. 4 ▸, are also seen in the 2 ≤ *x* ≤ 6 compounds (Karube *et al.*, 2016 ▸, 2017 ▸, 2018 ▸, 2020 ▸) and are understood to indicate the development of antiferromagnetic-like correlations on cooling. These antiferromagnetic correlations are known to serve as magnetic defects, as shown by the dramatic increase in spin wave damping observed in Co_8_Zn_8_Mn_4_ (Ukleev *et al.*, 2022 ▸). The increase in *w* over the same *T* range shows the gradual development of magnetic disorder in terms of the reduction of the radial correlation length, which itself provides a measure of the size of helical domains along the average direction of *q*_0_. This reduction in the radial correlation length could contribute to the apparent reduction in *A* between ∼80 and ∼40 K implied by our analysis, but we stress that a full treatment of the temperature dependence of *A* requires complete measurement of the scattering intensity distribution both within and normal to the detector plane. As mentioned above, it was not possible to measure the latter due the inherently short longitudinal magnetic correlation lengths, which cause the intensity distribution normal to the detector plane to extend far beyond the experimentally accessible range allowed by the cryomagnet windows. Therefore, further investigations designed to measure the full scattering intensity are needed for the accurate determination of the temperature dependence of *A*.

Turning to the fitted values of *w* shown in Fig. 4 ▸(*d*), they are seen to be always significantly larger than the instrumental resolution, allowing a simple estimation of the radial correlation length using ξ ≃ 1/*w*. We find that the radial correlation length near to *T*_c_ is ∼25 nm, *i.e.* of the order of 40 cubic unit-cell spacings, before it falls on cooling to ∼7 nm below *T*_g_, which is just above 10 cubic unit-cell spacings. It is notable that ξ is always shorter than λ, which indicates that the helical spin correlations are typically only short-range ordered. Such a tendency towards helical short-range order can reflect competing magnetic interactions on the mesoscale in this system, while we also note that, according to theory, defect-induced disorder can contribute further to the broadening of the helical Bragg peak (Utesov *et al.*, 2015 ▸).

The general behaviour observed in the ZFC temperature dependence of the *A*, *w* and *q*_0_ parameters in Co_6.75_Zn_6.75_Mn_6.5_ (*x* = 6.5) bears qualitative similarity to that observed in the 2 ≤ *x* ≤ 6 compounds (Karube *et al.*, 2016 ▸, 2017 ▸, 2018 ▸, 2020 ▸). In comparison with the lower-*x* compositions however, the magnetic disorder and disordering process on ZFC in the present *x* = 6.5 sample is clearly more pronounced. Firstly, by 80 K, the minority sample fraction of helical-like order showing up as SANS peaks at 100 K undergoes a full loss of orientational order by 80 K. The full loss of helical orientational order is not observed in any sample from 0 ≤ *x* ≤ 6 on ZFC down to base temperature. Secondly, this is followed by the strongest reduction in the radial correlation length amongst the studied compositions (Karube *et al.*, 2016 ▸, 2017 ▸, 2018 ▸, 2020 ▸). The present results thus extend to *x* = 6.5 the empirically observed inverse correlation between the increase in Mn concentration *x* and the stability of helical order at low *T* for 0 ≤ *x* ≤ 6. In addition, the present data imply that the *x* = 6.5 sample provides a future opportunity for detailed studies of an unusual *melting* of the helical correlations on *cooling* from *T*_c_ to *T*_g_, and potentially spiral spin liquid characteristics (Gao *et al.*, 2017 ▸).

#### 3.2.2. Skyrmion correlations in a finite magnetic field

**Figure 5:**
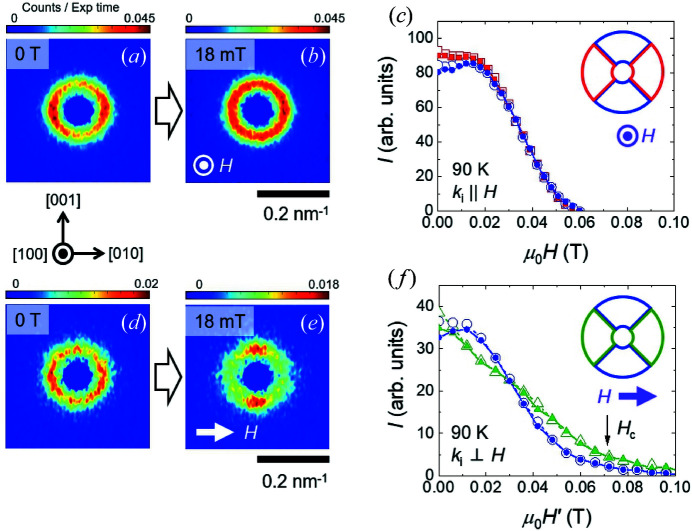
SANS patterns obtained in the **H** ∥ **k**_i_ geometry at ∼90 K, (*a*) in zero field and (*b*) at 18 mT. (*c*) The *H* dependence of the SANS intensities integrated over the 90° wide sector boxes indicated by the inset. Blue (red) symbols correspond to the sum of intensities in the two blue (red) 90° sectors. (*d*), (*e*) SANS patterns obtained at *H* = 0 and 18 mT, respectively, for the **H** ⊥ **k**_i_ geometry at 90 K. (*f*) The *H* dependence of the SANS intensities integrated over the 90° wide sector boxes indicated by the inset. Blue (green) symbols correspond to the sum of intensities in the two blue (green) 90° sectors. In panels (*c*) and (*f*), closed (open) symbols denote data obtained in an *H*-increasing (*H*-decreasing) scan. For the **H** ⊥ **k**_i_ geometry, the magnetic field scale is corrected for demagnetization effects and calibrated according to *H*′ = *H* × 3.0.

Next, we turn to measurements in a finite magnetic field. Figs. 5 ▸(*a*) and 5 ▸(*b*) show SANS patterns obtained in the **H** ∥ **k**_i_ geometry at 90 K, and in zero field and at 18 mT, respectively. The pattern in zero field is consistent with that shown in Fig. 3 ▸(*d*), where the SANS intensity forms a ring-like structure with more intensity on the right- and left-hand sides due to azimuthally broadened helical spots. Application of the magnetic field leads to a homogenization of the intensity distribution such that it forms a near isotropic intensity ring by 18 mT [Fig. 5 ▸(*b*)]. As will be supported from measurements in the **H** ⊥ **k**_i_ geometry, this *H*-driven rearrangement of the intensity is consistent with a field-induced transition of helical spin correlations into orientationally disordered skyrmion lattice correlations, as opposed to an *H*-driven disordering process.

Fig. 5 ▸(*c*) shows the magnetic field dependence of the summed SANS intensities within the two top/bottom (blue) 90° sectors and the two left/right (red) 90° sectors for the **H** ∥ **k**_i_ geometry. Closed (open) symbols denote *H*-increasing (*H*-decreasing) measurements with little intensity hysteresis observed. In zero field, the intensity in the red sector box pair is only 12 (2)% larger than that in the blue sector box pair, showing that the majority of the observed SANS intensity is due to the ring-like intensity component. The difference between the red and blue curves falls monotonically as the field is increased, eventually forming a fully azimuthally isotropic intensity distribution that survives until saturation.

Figs. 5 ▸(*d*)–5 ▸(*e*) show the SANS data obtained at 90 K in the **H** ⊥ **k**_i_ geometry, in zero field and at 18 mT. In this geometry, the pattern obtained at 18 mT shows the magnetic field to drive the partial rearrangement of the ring-like intensity in zero field so that there are two clear intensity maxima aligned with the vertical [001] direction. Since, in chiral cubic magnets, the skyrmion lattice forms with propagation vectors distributed in the plane perpendicular to the magnetic field, the emergence of additional top/bottom SANS intensity seen clearly in Fig. 5 ▸(*e*) corresponds to a hallmark signature for two-dimensional skyrmion lattice correlations (Mühlbauer *et al.*, 2009 ▸; Karube *et al.*, 2016 ▸). In Fig. 5 ▸(*f*) the formation of the skyrmion tubes aligned with **H** is manifested as a magnetic field range for which the combined intensity in the two top/bottom (blue) 90° sectors is maximally ∼20% larger than those of the two left/right (green) 90° sectors. On further increase in the field, the relative intensity of the green sector box pair becomes larger than that of the blue sector box pair before saturation. This can be understood in terms of the generally enhanced susceptibility of helical (or conical) spin correlations when the field is applied in the direction of propagation. Therefore, the SANS intensity due to helical spin correlations propagating relatively close to the applied field direction survives to higher fields compared with helical and skyrmion correlations propagating along directions far away from the direction of **H**.

**Figure 6:**
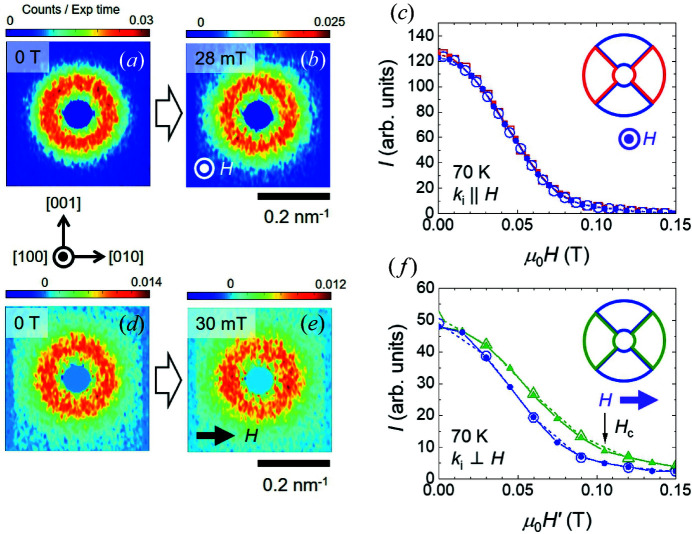
SANS patterns obtained in the **H** ∥ **k**_i_ geometry at ∼70 K, (*a*) in zero field and (*b*) at 28 mT. (*c*) The *H* dependence of the SANS intensities integrated over the 90° wide sector boxes indicated by the inset. Blue (red) symbols correspond to the sum of intensities in the two blue (red) 90° sectors. (*d*), (*e*) SANS patterns obtained at *H* = 0 and 30 mT, respectively, for the **H** ⊥ **k**_i_ geometry at 90 K. (*f*) The *H* dependence of the SANS intensities integrated over the 90° wide sector boxes indicated by the inset. Blue (green) symbols correspond to the sum of intensities in the two blue (green) 90° sectors. In panels (*c*) and (*f*), closed (open) symbols denote data obtained in an *H*-increasing (*H*-decreasing) scan. For the **H** ⊥ **k**_i_ geometry, the magnetic field scale is corrected for demagnetization effects and calibrated according to *H*′ = *H* × 3.0.

By 70 K, no magnetic-field-driven rearrangement of the SANS intensity is observed that is consistent with a conventional skyrmion formation signature in chiral magnets. Fig. 6 ▸ shows the relevant SANS data and data analysis at this temperature, which are analogous to those shown in Fig. 5 ▸ obtained at 90 K. In the **H** ∥ **k**_i_ geometry, Figs. 6 ▸(*a*) and 6 ▸(*b*), respectively, show the SANS patterns obtained in zero field and at 28 mT. In each case, the azimuthal distribution of the SANS intensity appears isotropic around the origin, with this confirmed at all fields in the *H* scans by the quantitative analysis presented in Fig. 6 ▸(*c*). For the **H** ⊥ **k**_i_ geometry, Figs. 6 ▸(*d*) and 6 ▸(*e*) show that the intensity ring in zero field evolves with increasing *H* so that there is slightly more intensity on the left- and right-hand sides at 30 mT. This is borne out by the quantitative analysis shown in Fig. 6 ▸(*f*), which shows that as *H* increases the SANS intensities extracted from the green left/right sector boxes [inset Fig. 6 ▸(*f*)] remain higher until saturation. Similar to the behaviour at 90 K, this can be understood in terms of the higher susceptibility for helical correlations propagating closer to the direction of **H**. Importantly, the analysis shows that, by 70 K, no standard signature of equilibrium skyrmion formation is detected, and the magnetic state is always predominantly three-dimensionally disordered. Further measurements confirm that this picture prevails down to the base temperature.

**Figure 7:**
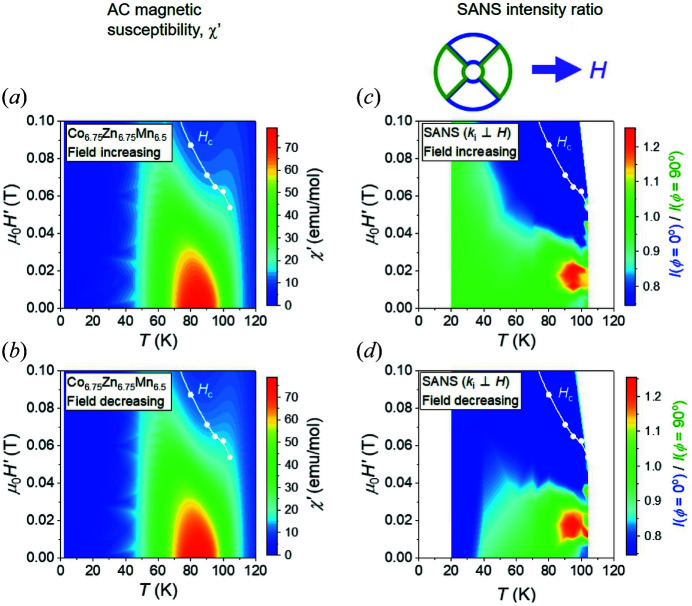
(*a*), (*b*) Magnetic phase diagrams inferred from a.c. susceptibility for (*a*) *H*-increasing and (*b*) *H*-decreasing scans at different temperatures. The colour scale denotes the size of χ′. (*c*), (*d*) Intensity phase diagrams inferred from SANS for (*c*) *H*-increasing and (*d*) *H*-decreasing scans at different temperatures. The colour scale denotes the ratio of summed SANS intensities obtained from integrating over the blue and green 90° wide sector box pairs indicated by the inset drawing above panel (*c*). In all panels, white symbols denote the upper critical field *H*_c_ which was determined to be the field where the slope of the *H*-dependent SANS intensity in the boxes parallel to the field [green boxes denoted *I*(φ = 90°)] decreases [see the black arrows in Figs. 5 ▸(*f*) and 6 ▸(*f*)]. Since estimates for *H*_c_ were difficult to obtain from the a.c. susceptibility data (due to its very smooth *H* dependence), the same values for *H*_c_ determined from the SANS data are plotted on colour maps shown in panels (*a*) and (*b*).

To summarize the *H*-dependent measurements on single-crystal Co_6.75_Zn_6.75_Mn_6.5_, in Fig. 7 ▸ we present phase diagrams constructed from the real part of the a.c. magnetic susceptibility χ′ [Figs. 7 ▸(*a*) and 7 ▸(*b*)] and SANS [Figs. 7 ▸(*c*) and 7 ▸(*d*)] data. Figs. 7 ▸(*a*) and 7 ▸(*b*) show phase diagrams constructed from, respectively, *H*-increasing and *H*-decreasing scans done at constant temperature. These two phase diagrams are very similar, and bear little resemblance to those of either low-*x* (Co_0.5_Zn_0.5_)_20−*x*_Mn_*x*_ compounds (Karube *et al.*, 2020 ▸) or archetypal chiral magnets such as MnSi (Mühlbauer *et al.*, 2009 ▸) and Cu_2_OSeO_3_ (Seki *et al.*, 2012 ▸). In the latter-mentioned systems, χ′ measurements are particularly useful for identifying the extent in *T* and *H* of different phases (Bauer & Pfleiderer, 2012 ▸; Birch *et al.*, 2020 ▸). In the present case, the most striking features are the *T*-smeared low-field transitions at *T*_c_ and *T*_g_, the vanishing of χ′ for *T* < *T*_g_ ≃ 50 K, and the monotonic *H* variation of χ′ in the region of *T*_g_ < *T* < *T*_c_(*H*). In contrast to clear signatures in χ′ revealing magnetic phase transition boundaries in chiral magnets like the *B*20s, no fine structure in χ′ is identifiable from the present sample, and none indicating skyrmion formation. Instead, χ′ is presumably dominated by the temperature evolution of the majority fraction of disordered helical spin correlations, and the spin-glass-like properties at low *T*.

In contrast, the signature for skyrmion formation shows up more clearly in phase diagrams constructed from SANS data obtained in the **H** ⊥ **k**_i_ geometry, for both *H*-increasing [Fig. 7 ▸(*c*)] and *H*-decreasing [Fig. 7 ▸(*d*)] scans at constant temperature. The colour scale describes the ratio of summed SANS intensities measured in the blue top/bottom sector boxes [denoted *I*(φ = 0°)] to that determined from the green left/right sector boxes [denoted *I*(φ = 90°)]. In both phase diagrams, and in the field-reversible region *T*_g_ < *T* < *T*_c_(*H*), the red portion of the colour plots indicates the excess of intensity in the top/bottom sectors, providing the clearest indication for the thermodynamic conditions under which skyrmions may form in the system. The location of this red portion of the phase diagram is similar to that for the conventional two-dimensional skyrmion *A* phase in chiral cubic magnets, namely occupying a narrow temperature region directly below *T*_c_ and at finite *H*. In quantitative terms, the thermal extent of the *A* phase in reduced temperature *T*/*T*_c_ is ∼0.16, which is comparable to that in Co_7_Zn_7_Mn_6_ (Karube *et al.*, 2018 ▸), but the intensity ratio between the blue and green pairs of integration sectors in the ‘skyrmion phase’ is only ∼1.2 and the majority of the SANS intensity nonetheless remains attributable to the disordered helical correlations. From this perspective, the skyrmion phase is largely suppressed in the present Co_6.75_Zn_6.75_Mn_6.5_ sample compared with lower *x* compositions. Apart from the minority skyrmion phase, disordered helical configurations dominate the phase diagram above *T*_g_ and for fields below saturation, with the green colours indicating ratios between blue and green sector box intensities that are closer to 1. Finally, in the region below *T*_g_, there is a pronounced irreversibility in the intensity ratio defined by the colour scale between the *H*-increasing and *H*-decreasing phase diagrams, consistent with that expected from a spin-glass-like regime.

### 3.3. Lorentz transmission electron microscopy

**Figure 8:**
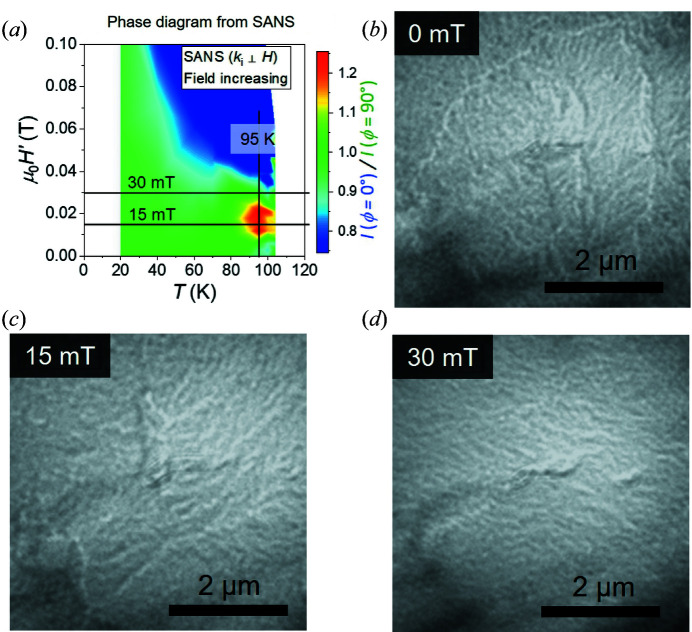
(*a*) The phase diagram deduced from the SANS data shown in Fig. 7 ▸(*c*). Lines and additional labels indicate *H* and *T* conditions under which the LTEM data shown in panels (*b*)–(*d*) were collected. (*b*)–(*d*) Overfocused LTEM images obtained from a Co_6.75_Zn_6.75_Mn_6.5_ lamella at 95 K. Data were obtained at (*b*) *H* = 0, (*c*) *H* = 15 mT and (*d*) *H* = 30 mT in an *H*-increasing scan after ZFC.

As it is one of the key experimental tools for studying helical and skyrmion spin textures in chiral magnets (Kanazawa *et al.*, 2017 ▸; Huang *et al.*, 2018 ▸; Tokura & Kanazawa, 2021 ▸), we employed LTEM with the aim of direct visualization of the magnetic textures below *T*_c_ in a thin plate sample of Co_6.75_Zn_6.75_Mn_6.5_. Fig. 8 ▸(*a*) shows the phase diagram of Fig. 7 ▸(*c*), with labels indicating the *T* and *H* conditions under which the LTEM images shown in Figs. 8 ▸(*b*)–8 ▸(*d*) were obtained. In all images, neither periodic magnetic textures nor (isolated) topological skyrmions can be identified. This indicates the magnetic contrast to be extremely weak, which is presumably due to the strong magnetic disorder, not only within the plane but also along the sample thickness. This contrasts with LTEM studies on lower-*x* compounds, including *x* = 6, where unambiguous imaging of helical order and skyrmions has been performed despite the existence of moderate magnetic disorder (Tokunaga *et al.*, 2015 ▸; Morikawa *et al.*, 2017 ▸; Karube *et al.*, 2017 ▸, 2018 ▸).

### 3.4. Magnetic diffuse neutron scattering

Finally, we turn to magnetic diffuse neutron scattering measurements from a Co_6.75_Zn_6.75_Mn_6.5_ powder sample taken using the D7 spectrometer at the ILL. With these measurements we explore magnetic neutron scattering at higher momentum transfers than afforded by SANS, which extend down to atomic length scales. In particular, we seek experimental evidence for persistent short-range magnetic correlations. Earlier work on β-Mn-type samples shows structured magnetic diffuse scattering to be a common experimental signature for short-range correlations of Mn moments on the 12*d* site (Stewart *et al.*, 2008 ▸, 2009 ▸, 2010 ▸; Stewart & Cywinski, 2009 ▸; Paddison *et al.*, 2013 ▸). The origin of the scattering is ascribed to the strong geometric frustration of interacting Mn moments on the 12*d* site with its frustration-inducing hyper-kagomé geometry. It is notable that qualitatively similar magnetic diffuse scattering was reported from measurements on the Co_7_Zn_7_Mn_6_ (*x* = 6) compound (Ukleev *et al.*, 2021 ▸). Since the average 12*d* occupation for *x* = 6 is Co:Zn:Mn = 0.2:7.0:4.8 (Nakajima *et al.*, 2019 ▸), it becomes clear that only partial average occupation of the 12*d* site by magnetic Mn ions is needed for magnetic diffuse scattering to show up. In this context it can be expected that a similar experimental signature is observed for the present *x* = 6.5 composition, since the average Mn content of the 12*d* site is only slightly higher, namely Co:Zn:Mn = 0.25:6.75:5.0 (Nakajima *et al.*, 2019 ▸).

**Figure 9:**
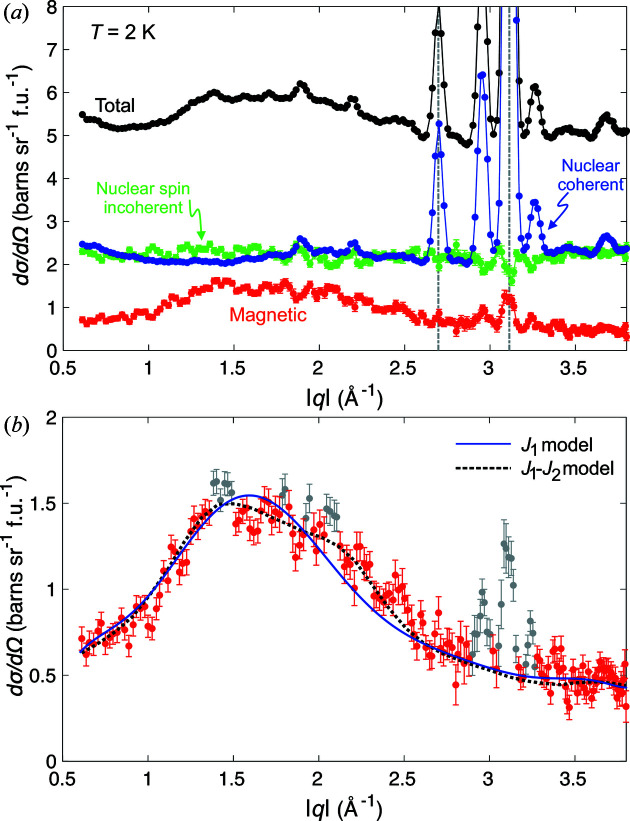
(*a*) Neutron diffuse scattering measurements as a function of modulus wavevector |*q*| from a Co_6.75_Zn_6.75_Mn_6.5_ powder sample at 2 K. The total observed scattering cross section is shown by black symbols. Using *xyz* polarization analysis, the nuclear coherent (blue symbols), nuclear spin-incoherent (green symbols) and magnetic (red symbols) scattering cross sections are extracted from the total scattering. Grey dashed lines indicate nuclear peak positions due to Al Bragg scattering from both the cryostat and sample container. (*b*) A closer look at the pure magnetic component of the total scattering cross section. Both grey and red symbols represent the entire measured profile. Grey symbols denote either clear or suspected Bragg scattering, and are excluded from the data set for the purposes of fitting the mean-field model to the data. Two fits of the mean-field model discussed in the text are shown. The blue solid line corresponds to a fit of a mean-field model that takes into account just a single nearest-neighbour antiferromagnetic interaction on the 12*d* site, *J*_1_ = −0.53 meV. The black dashed line shows the improved fit of the mean-field model when taking into account a second nearest-neighbour interaction *J*_2_. In this case the fitted parameters are *J*_1_ = −0.67 meV and *J*_2_ = 0.67 meV, indicating a probable combination of both antiferromagnetic and ferromagnetic interactions on the 12*d* site.

Using the *xyz* polarization analysis method on D7, the nuclear coherent, magnetic and nuclear spin-incoherent scattering cross sections are straightforwardly and cleanly isolated from the total scattering observed (Stewart *et al.*, 2009 ▸). Fig. 9 ▸(*a*) shows these various scattering cross sections measured from Co_6.75_Zn_6.75_Mn_6.5_ powder as a function of modulus wavevector |*q*| at 2 K and zero magnetic field. The nuclear spin-incoherent scattering intensity displays no |*q*| dependence as expected, while in the nuclear coherent scattering both structural diffraction peaks and diffuse scattering due to structural disorder are observed. Since the studied *q* range is too limited to extract detailed information on the origin of the structural diffuse scattering, in what follows we focus on the magnetic diffuse scattering.

Fig. 9 ▸(*b*) shows a closer view of the observed magnetic diffuse scattering cross section versus |*q*|. The scattering is composed largely of a peak that is broad in momentum transfer around |*q*| ≃ 1.7 Å^−1^, with additional weak peak-like features that are consistent with being due to the disordered helical correlations observed by SANS. For further data treatment and analysis of the magnetic diffuse scattering, we follow the same approach reported for *x* = 6 (Ukleev *et al.*, 2021 ▸). Namely, the weak peaks are removed and the remaining diffuse scattering is fitted to a mean-field model that accounts for the paramagnetic scattering due to interactions between Mn–Mn moments occupying the 12*d* site. Further technical details for the calculation method are reported by Ukleev *et al.* (2021 ▸). Here we mention that, for simplicity, similarly to the approach of Ukleev *et al.* (2021 ▸), an average crystal structure model is assumed, as well as a 12*d* site that is fully occupied by magnetic moments. The blue line in Fig. 9 ▸(*b*) shows the fit according to this minimal model that considers just a single nearest-neighbour antiferromagnetic exchange constant with fitted *J*_1_ = −0.53 meV, and a non-interacting temperature-dependent spin susceptibility. The reasonable fit of the model makes it plausible that the diffuse scattering is due to frustrated nearest-neighbour antiferromagnetic Mn–Mn interactions on the 12*d* site. The fitted value of *J*_1_ is essentially identical to that found for *x* = 6 (*J*_1_ = −0.54 meV), consistent with the observation that the diffuse scattering profiles from the two compounds are quantitatively similar.

Inclusion in the model of a second nearest-neighbour ferromagnetic interaction *J*_2_ leads to an improvement in the fit, as shown by the black dashed line in Fig. 9 ▸(*b*). The fit yields *J*_1_ = −0.67 meV and *J*_2_ = 0.67 meV, which have the same magnitude but opposite sign, thus implying that the proper description for the distribution of magnetic diffuse scattering involves a combination of competing antiferromagnetic (*J*_1_) and ferromagnetic (*J*_2_) interactions. While future studies on single-crystal samples are needed to quantify the interactions more rigorously, at the level of the data and analysis shown here, it is reasonable to conclude that the origin of magnetic diffuse scattering involves a major contribution from nearest-neighbour antiferromagnetic interactions between Mn moments on the 12*d* site.

## 4. Discussion

Our neutron scattering and magnetometry measurements reveal that the interactions and compositional disorder in the chiral magnet Co_6.75_Zn_6.75_Mn_6.5_ conspire to generate magnetically disordered phases at low temperature, over both atomic and mesoscopic length scales. As deduced in previous studies of Co–Zn–Mn compounds, mesoscale helices and skyrmions originate from the chiral magnetism of spins occupying the 8*c* site, involving mainly ferromagnetic Co ions (Karube *et al.*, 2016 ▸, 2017 ▸, 2018 ▸, 2020 ▸; Bocarsly *et al.*, 2019 ▸; Ukleev *et al.*, 2019 ▸, 2021 ▸). For 0 < *x* < 3, antiferromagnetic Mn fills in exclusively onto the geometrically frustrated 12*d* site, while for *x* > 3 Mn starts to fill in and replace Co on the 8*c* site too (Nakajima *et al.*, 2019 ▸). As borne out by the sharp suppression of *T*_c_, the emergence of spin glass behaviour and the tendencies towards magnetic disorder, adding Mn is generally unfavourable for mesoscale chiral magnetism. At the same time, however, the reduced temperature (*T*/*T*_c_) extent of the high-temperature skyrmion phase progressively increases with *x* for the 3 < *x* < 6 compounds, implying that Mn substitution effectively enhances skyrmion phase stability within the phase diagram (Karube *et al.*, 2020 ▸). In the following, we discuss the origin of the disordered mesoscale magnetism and the suppression of the equilibrium high-temperature skyrmion phase in the *x* = 6.5 sample.

As seen in Fig. 1 ▸(*b*), compared with lower-*x* compounds, an important aspect of the present *x* = 6.5 system is that the characteristic temperatures *T*_c_ and *T*_g_ are closer together, which already indicates a closer competition between chiral magnetism on the one hand, and atomic-scale frustration and disorder on the other. In addition, the location of *T*_c_ itself is also important in relation to the characteristic temperatures *T*_H_ and *T*_L_, which bound the temperature range over which the helical *q* and degree of mesoscale disorder both increase on cooling for 3 < *x* < 6. Both of these effects are understood to reflect the developing role of correlations involving Mn moments on both the 8*c* and 12*d* sites (Karube *et al.*, 2020 ▸). For *x* = 6.5, *T*_c_ already lies between the values of *T*_H_ and *T*_L_ that would be expected for this composition according to an extrapolation of their lower *x* dependences. In turn, this implies that, when helical correlations begin to form, Mn spin correlations detrimental to long-range order are already present, and thus mesoscale magnetism that is largely only short-range ordered is observed, together with the near-complete suppression of the high-temperature skyrmion phase.

Overall, the data across all low-*x* compositions indicate that when the Mn spin correlation onset temperature *T*_H_ < *T*_c_, *i.e.* for 3 < *x* ≤ 6 where helical long-range order occurs on cooling below *T*_c_, progressive introduction of Mn enhances high-temperature skyrmion phase stability in terms of *T*/*T*_c_, despite a concomitant increase in mesoscale disorder. This tendency ends when *T*_c_ < *T*_H_, and pre-existing inter-site Mn spin correlations dominate the helical ordering process such that skyrmions can no longer form. We also stress that the suppression of the skyrmion phase for *x* = 6.5 could not be expected by merely examining the *x* dependence of *T*_c_ and *T*_g_ alone, with SANS measurements proving crucial to obtain the necessary microscopic picture.

Next we discuss the mesoscale magnetic disorder in Co_6.75_Zn_6.75_Mn_6.5_ (*x* = 6.5). As mentioned above, our SANS data show the mesoscale magnetism to be dominated always by a majority fraction of fully orientationally disordered helical spin correlations. On ZFC below *T*_c_, further magnetic disordering via the reduction of the radial correlation length takes place, which is quantitatively stronger than that observed in lower-*x* compounds, including the nearby composition Co_7_Zn_7_Mn_6_ (*x* = 6) (Karube *et al.*, 2018 ▸). While a detailed description of the ZFC helical disordering process presents an interesting challenge for theory, we point out that a key ingredient must lie with the degree of Co–Mn mixing on the 8*c* site. According to neutron diffraction, the average 8*c* occupation for Co_6.75_Zn_6.75_Mn_6.5_ is Co:Zn:Mn = 6.5:0:1.5, while for Co_7_Zn_7_Mn_6_ it is Co:Zn:Mn = 6.8:0:1.2 (Nakajima *et al.*, 2019 ▸). In general, for the 8*c* site containing a majority Co/minority Mn mixture, the ferromagnetic Co exchange field can be expected to drive a ferromagnetic alignment of small ordered Mn moments just below *T*_c_, so that these Mn moments do indeed contribute to helically modulating spin correlations. This picture has been confirmed in both Co_8_Zn_8_Mn_4_ (Ukleev *et al.*, 2019 ▸) and Co_7_Zn_7_Mn_6_ (Ukleev *et al.*, 2021 ▸) by element-selective resonant X-ray scattering measurements. On cooling below *T*_H_, the development of couplings between growing 8*c* Mn moments and erstwhile magnetically disordered 12*d* Mn moments competes with the Co exchange field. This will result in disordering due to the random deviation of 8*c* Mn moments on cooling, as they tend towards a disordered ground state native to very Mn-rich compounds. In this qualitative picture, the tendency towards increasingly disordered helical textures will clearly be stronger in Mn-richer Co_6.75_Zn_6.75_Mn_6.5_ compared with lower-*x* compounds, consistent with SANS observations (Karube *et al.*, 2016 ▸, 2018 ▸). Further contributions to the mesoscale disorder are also expected to arise due to the compositional disorder which will modify locally the atomic scale interactions such as the DMI. Theory shows that such a bond disorder can distort helical spirals and contribute to the broadening of the SANS peaks (Utesov *et al.*, 2015 ▸).

Concerning topological skyrmions, our SANS measurements provide convincing evidence for the formation of conventional quasi-two-dimensional, yet in-plane orientationally disordered, static skyrmion correlations in a minority fraction of the Co_6.75_Zn_6.75_Mn_6.5_ sample. These correlations are stable in a portion of the phase diagram that is similar to the conventional skyrmion phase just below *T*_c_ in chiral magnets, and their presence is not obviously identifiable from the magnetometry and LTEM data at hand. An explanation as to why skyrmions exist only as a minority phase in Co_6.75_Zn_6.75_Mn_6.5_ is difficult to ascertain unambiguously, but it may lie with randomly distributed local anisotropies induced by correlated structural disorder. Such local anisotropies could cause a local fine tuning of interactions so that pinning and disorder effects are less important, and a fraction of zero-field helical correlations are able to transform into skyrmions near to *T*_c_.

Clearly in Co_6.75_Zn_6.75_Mn_6.5_ we obtain no experimental evidence for a low-temperature skyrmion phase far from *T*_c_, which contrasts with the observation of a second, low-temperature, equilibrium skyrmion phase in Co_7_Zn_7_Mn_6_ (Karube *et al.*, 2018 ▸). Since no low-temperature skyrmion phase is found in the lower-*x* neighbouring composition Co_8_Zn_8_Mn_4_ (Karube *et al.*, 2016 ▸, 2020 ▸) either, this makes Co_7_Zn_7_Mn_6_ special amongst the Co–Zn–Mn compounds. From comparative muon spin relaxation studies of Co_8_Zn_8_Mn_4_ and Co_7_Zn_7_Mn_6_, it was argued that a key ingredient for low-temperature skyrmion phase stability in Co_7_Zn_7_Mn_6_ is the low-temperature frustration-induced fluctuations of the 12*d* Mn moments (Ukleev *et al.*, 2021 ▸). According to the muon data, the associated density of magnetic fluctuations in the muon time window (typically the megahertz range) becomes particularly more pronounced at low temperature in Co_7_Zn_7_Mn_6_ compared with Co_8_Zn_8_Mn_4_. Although no equivalent muon data exist for Co_6.75_Zn_6.75_Mn_6.5_, it can be expected that megahertz fluctuations are also significant at low temperature in this system due to the higher Mn content. Thus, while the density of frustration-induced magnetic fluctuations is simply too low to assist with low-temperature skyrmion phase stabilization in Co_8_Zn_8_Mn_4_, in Co_6.75_Zn_6.75_Mn_6.5_ their density is likely to be at least comparable to that of Co_7_Zn_7_Mn_6_, but insufficient to stabilize skyrmions out of the strongly disordered low-temperature helical background. In general, muon spin relaxation experiments detect pronounced megahertz magnetic dynamics in and around the skyrmion phases of a number of chiral magnets (Hicken, Holt *et al.*, 2021 ▸; Hicken, Wilson *et al.*, 2021 ▸; Ukleev *et al.*, 2021 ▸). This makes the development of theoretical models for their origin at low temperature important, in order to test suggestions that they provide a route towards skyrmion phase stability.

Finally, we comment on the future perspectives for neutron scattering from quantum materials such as Co_6.75_Zn_6.75_Mn_6.5_. In the present system, we observe both structural and magnetic correlations with short- and long-range ordered character, over a broad range of momentum transfer that includes the conventional SANS range. It is clear that in complex quantum materials such as the Co–Zn–Mn intermetallics the eventual development of accurate theoretical models requires comprehensive measurement of structural and magnetic correlations over multiple length scales. While in principle the relevant scattering data can be pieced together from measurements done on different instruments covering the different dynamic ranges, for reasons of absolute scaling of scattering intensities and the avoidance of systematic uncertainties it is desirable to develop instrumentation capable of providing insight on the equal time correlations over a continuous extended range of momentum transfer. The data obtained from such instruments will facilitate the construction of atomistic theoretical models for multiple-length-scale magnets (such as Co–Zn–Mn compounds), which are needed for describing simultaneously the existence of magnetic long- and short-range order on both mesoscopic and atomic scales.

## 5. Conclusions

Using small-angle neutron scattering, magnetometry, Lorentz transmission electron microscopy and magnetic diffuse neutron scattering, we have investigated the microscopic helical and skyrmion correlations in the chiral magnet Co_6.75_Zn_6.75_Mn_6.5_ (*T*_c_ ≃ 102 K). SANS experiments reveal that, below *T*_c_, the mesoscopic phase diagram is dominated by orientationally disordered helical correlations that are largely unresponsive to applied magnetic fields below the saturation field. Close to *T*_c_, a minority fraction of helical correlations in the sample undergo a magnetic-field-driven transformation into conventional two-dimensional skyrmion correlations. The signature for skyrmion formation is not obviously observed in magnetometry or LTEM measurements, highlighting the power of SANS to reveal the existence of skyrmions in this particular case, due its ability to probe the entire sample volume.

The characteristically strong magnetic disorder observed on both mesoscopic and atomic length scales is argued to arise from the interplay between compositional disorder, in particular the random mixing of magnetically distinct Co and Mn moments on the 8*c* Wyckoff site, and the magnetic frustration of Mn moments occupying the 12*d* Wyckoff site. While short-range magnetic correlations due to 12*d* frustrated Mn–Mn interactions lead to the observed magnetic diffuse scattering on atomic length scales, the mesoscale order observed by SANS is due to modulating moments on the 8*c* site. To explain the unusual temperature-dependent disordering process of helically modulating 8*c* moments, we invoke a temperature-dependent coupling between Mn moments on the 12*d* and 8*c* sites that grows on cooling and promotes increasing mesoscale magnetic disorder due to random reorientations of erstwhile helically modulating 8*c* Mn moments. It is also likely that this strong tendency towards magnetic disorder on cooling prevents the stability of a second, low-temperature, skyrmion phase in Co_6.75_Zn_6.75_Mn_6.5_, which is different from what is observed in the nearby composition Co_7_Zn_7_Mn_6_.

Our study of Co_6.75_Zn_6.75_Mn_6.5_ also highlights a general need for neutron instrumentation that can provide continuous access to an extended range of momentum transfer that includes the SANS range, so that the magnetic correlations in multiple-length-scale magnets like the Co–Zn–Mn family can be measured comprehensively, thus facilitating the development of appropriate theoretical models.

## References

[bb1] Bauer, A. & Pfleiderer, C. (2012). *Phys. Rev. B*, **85**, 214418.

[bb2] Birch, M. T., Moody, S. H., Wilson, M. N., Crisanti, M., Bewley, O., Štefančič, A., Balakrishnan, G., Fan, R., Steadman, P., Alba Venero, D., Cubitt, R. & Hatton, P. D. (2020). *Phys. Rev. B*, **102**, 104424.

[bb3] Bocarsly, J. D., Heikes, C., Brown, C. M., Wilson, S. D. & Seshadri, R. (2019). *Phys. Rev. Mater.* **3**, 014402. 10.1103/PhysRevMaterials.3.014402 PMC11494682 39439577

[bb4] Bordács, S., Butykai, A., Szigeti, B. G., White, J. S., Cubitt, R., Leonov, A. O., Widmann, S., Ehlers, D., von Nidda, H. K., Tsurkan, V., Loidl, A. & Kézsmárki, I. (2017). *Sci. Rep.* **7**, 7584. 10.1038/s41598-017-07996-x PMC5548730 28790441

[bb5] Dewhurst, C. D. (2003). *GRASP User Manual*. Technical Report No. ILL03DE01T. Institut Laue–Langevin, Grenoble, France. https://www.ill.eu/fileadmin/user_upload/ILL/3_Users/Scientific_groups/Large_Scale_Structures/Grasp/Download/grasp_manual.pdf.

[bb6] Gao, S., Zaharko, O., Tsurkan, V., Su, Y., White, J. S., Tucker, G. S., Roessli, B., Bourdarot, F., Sibille, R., Chernyshov, D., Fennell, T., Loidl, A. & Rüegg, C. (2017). *Nat. Phys.* **13**, 157–161.

[bb7] Henderson, M. E., Beare, J., Sharma, S., Bleuel, M., Clancy, P., Cory, D. G., Huber, M. G., Marjerrison, C. A., Pula, M., Sarenac, D., Smith, E. M., Zhernenkov, K., Luke, G. M. & Pushin, D. A. (2021). *Materials*, **14**, 4689. 10.3390/ma14164689 PMC8399547 34443211

[bb8] Hicken, T. J., Holt, S. J. R., Franke, K. J. A., Hawkhead, Z., Štefančič, A., Wilson, M. N., Gomilšek, M., Huddart, B. M., Clark, S. J., Lees, M. R., Pratt, F. L., Blundell, S. J., Balakrishnan, G. & Lancaster, T. (2021). *Phys. Rev. Res.* **2**, 032001.

[bb9] Hicken, T. J., Wilson, M. N., Franke, K. J. A., Huddart, B. M., Hawkhead, Z., Gomilšek, M., Clark, S. J., Pratt, F. L., Štefančič, A., Hall, A. E., Ciomaga Hatnean, M., Balakrishnan, G. & Lancaster, T. (2021). *Phys. Rev. B*, **103**, 024428.

[bb10] Hori, T., Shiraish, H. & Ishii, Y. (2007). *J. Magn. Magn. Mater.* **310**, 1820–1822.

[bb11] Huang, P., Jayaraman, R., Mancini, G. F., Kruchkov, A., Cantoni, M., Murooka, Y., Latychevskaia, T., McGrouther, D., Baldini, E., White, J. S., Magrez, A., Giamarchi, T., Carbone, F. & Rønnow, H. M. (2018). *Microsc. Microanal.* **24**, 932–933.

[bb12] Kanazawa, N., Seki, S. & Tokura, Y. (2017). *Adv. Mater.* **29**, 1603227. 10.1002/adma.201603227 28306166

[bb13] Karube, K., White, J. S., Morikawa, D., Bartkowiak, M., Kikkawa, A., Tokunaga, Y., Arima, T., Rønnow, H. M., Tokura, Y. & Taguchi, Y. (2017). *Phys. Rev. Mater.* **1**, 074405.

[bb14] Karube, K., White, J. S., Morikawa, D., Dewhurst, C. D., Cubitt, R., Kikkawa, A., Yu, X. Z., Tokunaga, Y., Arima, T., Rønnow, H. M., Tokura, Y. & Taguchi, Y. (2018). *Sci. Adv.* **4**, eaar7043. 10.1126/sciadv.aar7043 PMC6140611 30225364

[bb15] Karube, K., White, J. S., Reynolds, N., Gavilano, J. L., Oike, H., Kikkawa, A., Kagawa, F., Tokunaga, Y., Rønnow, H. M., Tokura, Y. & Taguchi, Y. (2016). *Nat. Mater.* **15**, 1237–1242. 10.1038/nmat4752 27643728

[bb16] Karube, K., White, J. S., Ukleev, V., Dewhurst, C. D., Cubitt, R., Kikkawa, A., Tokunaga, Y., Rønnow, H. M., Tokura, Y. & Taguchi, Y. (2020). *Phys. Rev. B*, **102**, 064408.

[bb17] Kruchkov, A. J., White, J. S., Bartkowiak, M., Živković, I., Magrez, A. & Rønnow, H. M. (2018). *Sci. Rep.* **8**, 10466. 10.1038/s41598-018-27882-4 PMC6041276 29992965

[bb18] Kurumaji, T., Nakajima, T., Ukleev, V., Feoktystov, A., Arima, T., Kakurai, K. & Tokura, Y. (2017). *Phys. Rev. Lett.* **119**, 237201. 10.1103/PhysRevLett.119.237201 29286691

[bb19] Morikawa, D., Yu, X. Z., Karube, K., Tokunaga, Y., Taguchi, Y., Arima, T. & Tokura, Y. (2017). *Nano Lett.* **17**, 1637–1641. 10.1021/acs.nanolett.6b04821 28135106

[bb20] Moskvin, E., Grigoriev, S., Dyadkin, V., Eckerlebe, H., Baenitz, M., Schmidt, M. & Wilhelm, H. (2013). *Phys. Rev. Lett.* **110**, 077207. 10.1103/PhysRevLett.110.077207 25166404

[bb21] Mühlbauer, S., Binz, B., Jonietz, F., Pfleiderer, C., Rosch, A., Neubauer, A., Georgii, R. & Böni, P. (2009). *Science*, **323**, 915–919. 10.1126/science.1166767 19213914

[bb22] Mühlbauer, S., Honecker, D., Périgo, A., Bergner, F., Disch, S., Heinemann, A., Erokhin, S., Berkov, D., Leighton, C., Eskildsen, M. R. & Michels, A. (2019). *Rev. Mod. Phys.* **91**, 015004.

[bb23] Nakajima, T., Karube, K., Ishikawa, Y., Yonemura, M., Reynolds, N., White, J. S., Rønnow, H. M., Kikkawa, A., Tokunaga, Y., Taguchi, Y., Tokura, Y. & Arima, T. (2019). *Phys. Rev. B*, **100**, 064407.

[bb24] Nakamura, H., Yoshimoto, K., Shiga, M., Nishi, M. & Kakurai, K. (1997). *J. Phys. Condens. Matter*, **9**, 4701–4728.

[bb25] Paddison, J. A. M., Stewart, J. R., Manuel, P., Courtois, P., McIntyre, G. J., Rainford, B. D. & Goodwin, A. L. (2013). *Phys. Rev. Lett.* **110**, 267207. 10.1103/PhysRevLett.110.267207 23848920

[bb26] Preißinger, M., Karube, K., Ehlers, D., Szigeti, B., Krug von Nidda, H.-A., White, J. S., Ukleev, V., Rønnow, H. M., Tokunaga, Y., Kikkawa, A., Tokura, Y., Taguchi, Y. & Kézsmárki, I. (2021). *NPJ Quantum Mater.* **6**, 65.

[bb27] Puphal, P., Pomjakushin, V., Kanazawa, N., Ukleev, V., Gawryluk, D. J., Ma, J., Naamneh, M., Plumb, N. C., Keller, L., Cubitt, R., Pomjakushina, E. & White, J. S. (2020). *Phys. Rev. Lett.* **124**, 017202. 10.1103/PhysRevLett.124.017202 31976692

[bb28] Seki, S., Yu, X. Z., Ishiwata, S. & Tokura, Y. (2012). *Science*, **336**, 198–201. 10.1126/science.1214143 22499941

[bb29] Stewart, J. R., Andersen, K. H. & Cywinski, R. (2008). *Phys. Rev. B*, **78**, 014428.

[bb30] Stewart, J. R. & Cywinski, R. (2009). *J. Phys. Condens. Matter*, **21**, 124216. 10.1088/0953-8984/21/12/124216 21817458

[bb31] Stewart, J. R., Deen, P. P., Andersen, K. H., Schober, H., Barthélémy, J.-F., Hillier, J. M., Murani, A. P., Hayes, T. & Lindenau, B. (2009). *J. Appl. Cryst.* **42**, 69–84.

[bb32] Stewart, J. R., Hillier, A. D., Hillier, J. M. & Cywinski, R. (2010). *Phys. Rev. B*, **82**, 144439.

[bb33] Stewart, J. R., Rainford, B. D., Eccleston, R. S. & Cywinski, R. (2002). *Phys. Rev. Lett.* **89**, 186403. 10.1103/PhysRevLett.89.186403 12398623

[bb34] Takagi, R., White, J. S., Hayami, S., Arita, R., Honecker, D., Rønnow, H. M., Tokura, Y. & Seki, S. (2018). *Sci. Adv.* **4**, eaau3402. 10.1126/sciadv.aau3402 PMC6239426 30456302

[bb35] Tokunaga, Y., Yu, X. Z., White, J. S., Rønnow, H. M., Morikawa, D., Taguchi, Y. & Tokura, Y. (2015). *Nat. Commun.* **6**, 7638. 10.1038/ncomms8638 PMC4506512 26134284

[bb36] Tokura, Y. & Kanazawa, N. (2021). *Chem. Rev.* **121**, 2857–2897. 10.1021/acs.chemrev.0c00297 33164494

[bb37] Ukleev, V., Karube, K., Derlet, P. M., Wang, C. N., Luetkens, H., Morikawa, D., Kikkawa, A., Mangin-Thro, L., Wildes, A. R., Yamasaki, Y., Yokoyama, Y., Yu, L., Piamonteze, C., Jaouen, N., Tokunaga, Y., Rønnow, H. M., Arima, T., Tokura, Y., Taguchi, T. & White, J. S. (2021). *NPJ Quantum Mater.* **6**, 40.

[bb50] Ukleev, V., Pschenichnyi, K. A., Utesov, O., Karube, K., Mühlbauer, S., Cubitt, R., Tokura, Y., Taguchi, Y., White, J. S. & Grigoriev, S. V. (2022). *Phys. Rev. Res.* **4**, 023239.

[bb38] Ukleev, V., Yamasaki, Y., Morikawa, D., Karube, K., Shibata, K., Tokunaga, Y., Okamura, Y., Amemiya, K., Valvidares, M., Nakao, H., Taguchi, Y., Tokura, Y. & Arima, T. (2019). *Phys. Rev. B*, **99**, 144408.

[bb39] Utesov, O. I., Sizanov, A. V. & Syromyatnikov, A. V. (2015). *Phys. Rev. B*, **92**, 125110.

[bb40] White, J. S., Cubitt, R., Dewhurst, C. D., Karube, K. & Rønnow, H. M. (2017). *Search for New Skyrmion States in Co_6.75_Zn_6.75_Mn_6.5_.* Institut Laue–Langevin (ILL). https://doi.org/10.5291/ILL-DATA.5-42-443.

[bb41] White, J. S., Cubitt, R., Dewhurst, C. D., Karube, K., Rønnow, H. M. & Ukleev, V. (2019). *A Frustration-Induced Chiral Spin Liquid in Co_6.75_Zn_6.75_Mn_6.5_?* Institut Laue–Langevin (ILL). https://doi.org/10.5291/ILL-DATA.5-42-496.

[bb42] White, J. S., Karube, K., Rønnow, H. M. & Wildes, A. (2018). *From Chiral Helimagnet to Frustrated Spin Liquid by Tuning the Mn Concentration in Co_x_Zn_y_Mn_z_ Alloys.* Institut Laue–Langevin (ILL). https://doi.org/10.5291/ILL-DATA.5-32-855.

[bb43] Xie, W., Thimmaiah, S., Lamsal, J., Liu, J., Heitmann, T. W., Quirinale, D., Goldman, A. I., Pecharsky, V. & Miller, G. J. (2013). *Inorg. Chem.* **52**, 9399–9408. 10.1021/ic4009653 23909791

